# Removing bleaching artifacts from voltage sensitive dye recordings with ICA

**DOI:** 10.1186/1471-2202-14-S1-P222

**Published:** 2013-07-08

**Authors:** Elham Fathiazar, Gerrit Hilgen, Jutta Kretzberg

**Affiliations:** 1 Computational Neuroscience, University of Oldenburg, Oldenburg, 26111, Germany

## 

Voltage sensitive dye (VSD) imaging of multiple neurons becomes one of the most promising up-to-date methods to investigate neuronal network activity. However, optical imaging signals are often superimposed by noise and artifacts. Hence, post-processing methods are needed to overcome this corruption and separate neuronal activity from other signals. One of the significant artifacts in VSD imaging is bleaching, a decrease of the optical signal while the recorded signal of the local field potentials remains unchanged [1]. In this study we used independent component analysis (ICA) in comparison to principal component analysis (PCA) and detrend method to separate the neuronal VSD signals from bleaching artifacts. ICA is a blind source separation method that has been used in many different approaches such as to recover action potentials of neurons in multiple-detector optical recordings [3]. We used the ICA-DTU Toolbox [http://www2.imm.dtu.dk/pubdb/views/publication_details.php?id=4043] with maximum likelihood formulation to identify neuronal signals and the effect of bleaching.

**Figure 1:**
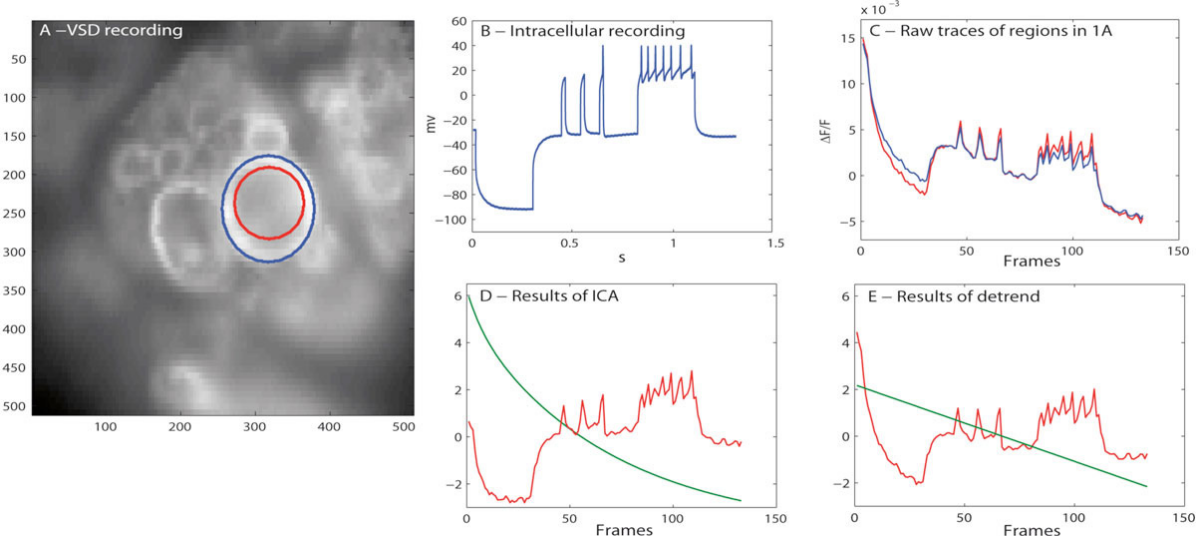
**VSD and intracellular recording of leech ganglion and analysis results**.

Experimentally, we recorded from Retzius cells of an isolated midbody ganglion of the electrophysiologically well- characterized leech nervous system. VSD imaging (Figure A) based on a new VSD dye with a very good signal-to-noise ratio [2] was combined with simultaneous intracellular recording of the membrane potential (Figure B) and electrical stimulation. VSD signals obtained from the region of the Retzius cell body (Figure 1A and 1C, red) and from a slightly larger region (Figure 1A and 1C, blue) were used as input signals to ICA. Of the two components returned by the algorithm one decreased exponentially, while the other resembled neuronal activity. Figure 1D shows an exponential fit of the component representing bleaching (green), and the estimated neuronal activity (red). Taking the lower sampling rate of the VSD signal (100 Hz vs 10,000 Hz) into account, this estimate reflects the dynamics of the intracellular recording (1B) well. While PCA was not able to separate bleaching from neuronal signals (not shown), the detrend method also yielded good results (Figure 1E). This method fits a piecewise linear line (1E, green) to the VSD signal, representing bleaching effect. However, the difference (1E, red) between the original trace and this line represents the graded de-and hyperpolarisations of the membrane potential seen in the intracellular recording (1B) less well than the ICA result (1D, red).
